# Etonogestrel contraceptive implant failure in a woman taking rifampin: a case report

**DOI:** 10.1186/s40834-022-00172-1

**Published:** 2022-05-05

**Authors:** Tesfaye H. Tufa, Abraham Fessehaye, Ferid A. Abubeker

**Affiliations:** Department of Obstetrics and Gynecology, St. Paul’s Hospital Millennium Medical College, Addis Ababa, Ethiopia

**Keywords:** Etonogestrel implant, Contraceptive failure, Anti-tuberculosis, Rifampin, Unintended pregnancy, Case report

## Abstract

**Background:**

The etonogestrel subdermal implant is the most efficacious hormonal contraceptive currently available and provides 99.7% effective contraception. However, similar to other hormonal contraception, its effectiveness is compromised with the use of cytochrome P450 inducing drugs resulting in an unplanned pregnancy. Despite this risk, little is known about the outcome of concomitant use of rifampin and contraceptive implants.

**Case presentation:**

A 24-year-old woman was provided with an etonogestrel implant in September 2018. In July 2020, she was started with rifampin based anti-tuberculosis for tuberculosis of the lymph nodes. In December 2020, she presented to the family planning clinic of St. Paul’s Hospital Millennium Medical college with a diagnosis of failed implant and second-trimester pregnancy at a gestational age of 19 weeks. The etonogestrel implant was removed and the patient was linked to antenatal care follow up.

**Conclusion:**

Concomitant use of hepatic cytochrome P450 enzyme-inducing medications with certain hormonal contraceptives may reduce effectiveness resulting in unintended pregnancy. Women should be given detailed counseling about the potential for drug interactions and a multidisciplinary approach with consultation or referral to reproductive health specialists is crucial for optimal management of women who are at increased risk of contraceptive failure and unintended pregnancy.

## Introduction

Contraceptive implants are among the most effective long-acting reversible contraceptive (LARC) methods [1]. Nexplanon® is a progestin-only, single-rod contraceptive implant containing 68 mg of etonogestrel placed in the inner side of the non-dominant upper arm [2]. The mechanism of action is mainly through suppression of ovulation augmented by increased cervical mucus viscosity that hinders the passage of spermatozoa [3]. Etonogestrel implant is the most efficacious hormonal contraceptive currently available and provides 99.7% effective contraception for up to three years [4, 5].

Cytochrome P450 enzyme (CYP450) inducing medications are reported to be one of the reasons for the failure of this highly effective contraceptive method. For example, rifampin, a highly effective anti-Tuberculosis (TB) drug, induces activation of CYP450. Cytochrome P450 in turn reduces contraception effectiveness by increasing serum clearance and making the hormonal contraception less available in the serum to prevent pregnancy [6, 7]. Similarly, other CYP450 enzyme inducing medications such as carbamazepine and efavirenz had resulted in a failure of etonogestrel implant [8, 9].

In this report, we present a case of failed etonogestrel implant that highlights the importance of client counseling about the possibility of drug interactions between hormonal contraception and rifampin-based anti-TB medications.

## Case presentation

In September 2018, a 24-year-old woman had an uncomplicated vaginal delivery at St. Paul’s Hospital Millennium Medical College (SPHMMC). Following her delivery, she was counseled on possible options of contraception by the attending doctor and opted for etonogestrel implant since she wanted to delay pregnancy for a minimum of three years. After reviewing her medical eligibility and excluding contraindications, a trained provider placed Nexplanon® (Merck & Co. Inc., Whitehouse Station, NJ) in the left arm as per insertion protocol. She noticed a reduction in the frequency and amount of menstrual bleeding after the first year of use. Otherwise, there were no side effects of the contraceptive device reported by the patient.

In July 2020, she was started on anti-TB drugs for the diagnosis of TB lymphadenitis. The treatment regimen consisted of rifampin, isoniazid, pyrazinamide, and ethambutol once daily for two months followed by a four-month course of daily rifampin and isoniazid.

During the initiation of anti-TB treatment, she claims that she was reassured by the health care provider about the effectiveness of etonogestrel implant and started the treatment without being informed about the possibility of drug interactions and unintended pregnancy. She was not advised to change the contraception method or use an additional method.

In November 2020, she went to a nearby health center after missing her menstrual period for four consecutive months. A pregnancy test was positive for which she was referred to SPHMMC with a diagnosis of second-trimester pregnancy and failed etonogetrel implant. Upon arrival to SPHMMC in December 2020, the patient had stable vital signs, and obstetric ultrasound showed a 19 weeks pregnancy with a normal fetal gross anatomical appearance. She expressed interest in continuing the pregnancy and was reassured about the lack of untoward effects from the contraceptive method on the fetus and the pregnancy. The etonogestrel implant was localized and removed from her left upper arm. She then attended regular antenatal care follow up.

## Discussion

The etonogestrel implant is one of the most effective LARC methods. In most healthy women it is more than 99% effective with a failure rate lower than permanent sterilization techniques [1, 5, 10]. Several factors have been implicated in the failure of implant contraceptives. In a review of more than 200 unintended pregnancies among women using etonogestrel implant, improper insertion was the most common reason for method failure accounting for 38% of cases whereas 4% of cases were due to drug interactions [11]. Another large study however showed that a large proportion of contraceptive failure (nearly 25%) was associated with intake of drugs that can potentially affect contraceptive efficacy [12].

Etonogestrel is metabolized by the hepatic CYP450 enzyme system. Drugs that induce CYP450 may increase the rate of hepatic degradation, leading to a lower bioavailability and potential loss of contraceptive effect [13]. For example, a pharmacologic study of women using etonogestrel implant showed a drop in the serum etonogestrel level below the threshold needed to inhibit ovulation after administration of carbamazepine, which is known to induce CYP450 enzymes [14]. Similar findings of drug interactions and failed implants were reported among women on other anti-epileptic, antiretroviral, and anti-TB drugs [7, 15, 16]. This patient was started on an anti-TB regimen containing rifampin, a known CYP450 enzyme inducer [13].

For women who are using or planning to use hormonal contraceptives, several considerations need to be made should they require treatment with medications that are CYP450 enzyme inducers. A multidisciplinary approach with consultation or referral to reproductive health specialists can play a significant role in optimal management. They need to receive detailed counseling about the potential for drug interaction. The discussion should include the use of other alternative methods. Both the World Health Organization and United States Center for Disease Control medical eligibility criteria recommend women on implants who need long-term treatment with rifampin to use other contraceptive methods [17, 18]. The United Kingdom (UK MEC) advises the use of additional barrier methods or a switch to other methods when the potential for drug interactions is a concern [19, 20]. The product label for Nexplanon® also indicates that rifampin reduces the effectiveness of etonogestrel contraceptive implants [21].

## Conclusion

This case report highlights the need for detailed contraceptive counseling for women with medical comorbidities. Health care providers should be aware of the possible reduced effectiveness of certain hormonal contraceptives due to concomitant use of hepatic CYP450 enzyme-inducing medications. Alternative contraceptive options should be offered in these cases. Thorough medical and contraceptive history with consultation or referral to reproductive health specialists is crucial for optimal management of women who are at increased risk of contraceptive failure and unintended pregnancy.

## Data Availability

All data materials related to the case report are included in the manuscript.

## Abbreviations

CYP450: Cytochrome P450
LARC: Long-Acting Contraceptives
MEC: Medical eligibility criteria
SPHMMC: St. Paul’s Hospital Millennium Medical College
TB: Tuberculosis
